# *Ex vivo* analysis of renal proximal tubular cells

**DOI:** 10.1186/s12860-015-0058-4

**Published:** 2015-03-25

**Authors:** David Legouis, Aurélien Bataille, Alexandre Hertig, Sophie Vandermeersch, Noémie Simon, Eric Rondeau, Pierre Galichon

**Affiliations:** INSERM, UMR-S1155, Paris, France; UPMC, Sorbonne Universités, Paris, 6 France; Urgences Néphrologiques et Transplantation Rénale, APHP, Paris, France

**Keywords:** Organ physiology, Experimental models, Cell phenotype, Primary cell cultures, In vivo, In vitro

## Abstract

**Background:**

Experimental models are inevitably a compromise between accurately reproducing a pathological situation and schematically simplifying it, which is intended to provide both relevance and conclusiveness. *In-vivo* models are very relevant, but multiple cell-types undergoing various changes may hinder the observation of individual molecular events.

**Results:**

Here, we describe a method for analyzing and isolating specific cell types from the kidney and studying the phenotype they have acquired *in vivo*. Using flow cytometry, immunofluorescence, and RT-PCR, we show that our method is suitable for studying and isolating proximal tubular cells with an anti Prominin-1 antibody. Kidneys are subjected to mechanical dissociation followed by flow-cytometry analysis. Hundreds of thousands of proximal tubular cells are then isolated by magnetic separation followed by direct analysis or primary cell culture. Using our method, we detect phenotypic changes in the proximal tubular cells after renal ischemia reperfusion, and we isolate the proximal tubular cells, with a purity over 80%.

**Conclusions:**

This method is efficient, quick, simple, and cheap, and should be useful for studying cell-type specific parameters after *in vivo* experimental studies. It is also a simple method to obtain a specific primary cell culture from any animal strain.

**Electronic supplementary material:**

The online version of this article (doi:10.1186/s12860-015-0058-4) contains supplementary material, which is available to authorized users.

## Background

Animal studies are performed in controlled environments, and provide an acceptable compromise for studying events that occur in human beings, providing clues for understanding when and where such events originally occur. For example, sufficient technological progress has now been achieved to make it possible to see, literally, when and where an acute kidney injury will induce patchy necrosis of tubular epithelial cells [1]. Obtaining mechanistic insight into why and how this occurs is another similarly important challenge: new drug discovery implies the ability to distinguish between the molecular causes and the biological effects in specific cell types. *In-vitro* studies clearly provide an important technical complement here [2-5]. The biological relevance of what is observed in a culture dish is, however, very uncertain, because the study environment is even more restricted. Furthermore, the cells are completely isolated from the source organ, as well as from the whole source animal. Not to mention the fact that immortalizing a cell lineage, something routinely performed to save time and money, may profoundly alter cell programming and how it responds to injury.

*Ex-vivo* study of the cell lysate of a whole organ subjected to some form of injury would obviously be uninformative, since it would mix many different cell types and could provide misleading observations or mask significant ones. Another approach would be to examine the population of interest after the injury has been imposed *in vivo* (for instance, looking at proximal tubular epithelial cells in the context of an acute kidney injury after trying to sort and capture living cells belonging to the population of interest *ex vivo)*. The limitations of this approach so far have been 1) the dissociation, using enzymes at 37°C, which can dramatically modify the cell phenotype (extracellular protein cleavage *and* transcriptomic changes may occur as early as 10 minutes after enzymatic exposure) [6-8], and 2) the heterogeneity of the dissociated cell suspension which, as far as we know, was always cultured before being studied (except for interstitial cells: leucocytes and fibroblasts) [9].

**Figure 1:**
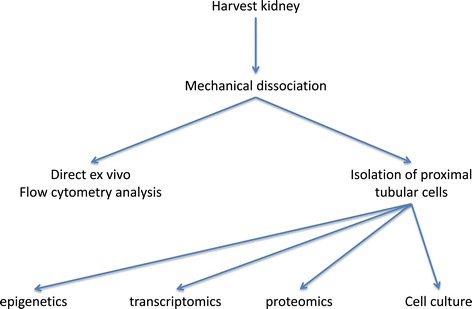
**Flowchart of the method detailing the three applications of dissociated cells: direct analysis, isolation of a subpopulation of cells for subsequent analysis, or for primary cell culture.**

Here, we describe a fast, low-cost method that keeps the cells alive and does not necessitate costly equipment, that can be used to extract thousands of intact proximal tubular cells from one or a few fresh kidneys, making it possible to carry out *ex vivo* and extemporaneous quantification of multiple molecular pathways or cell-type specific selection for subsequent analysis or culture (Figure 1).

## Results and discussion

### Instantaneous, enzyme-free preparation of a cellular suspension from whole kidneys

Immediately after being harvested from adult C57bl6/J mice, each kidney was immersed in dissociating buffer, chopped and dissociated using the GentleMACS cell dissociator (Miltenyl Biotec, California, USA) for 2 minutes at 4°C, with no added enzymes. This protocol, which takes no more than 10 minutes total, is detailed in the Methods section and in Table 1.

**Table 1:** **Protocol for direct dissociation of a whole kidney into a cell suspension**

1.	Prepare sterile dissociation buffer (4°C): PBS 1×, 0.5% bovine serum albumin, and 2 mM EDTA.
2.	Immerse the kidney in 1 mL of this dissociation buffer
3.	Remove the capsule and roughly chop the kidney with a surgical blade
4.	Transfer the solution obtained into GentleMACS C-tubes.
5.	Use the brain_03 and then the spleen_04 program of the GentleMACS dissociator.
6.	Pass the solution obtained through a 30-μm sieve.
7.	Rinse with 4 mL of dissociation buffer.
8.	Centrifuge at 500 g for10 minutes.
9.	Discard the supernatant.
10	Resuspend cell pellet in 180 μL of dissociating buffer.

### Debris exclusion prior to cell analysis

**Figure 2:**
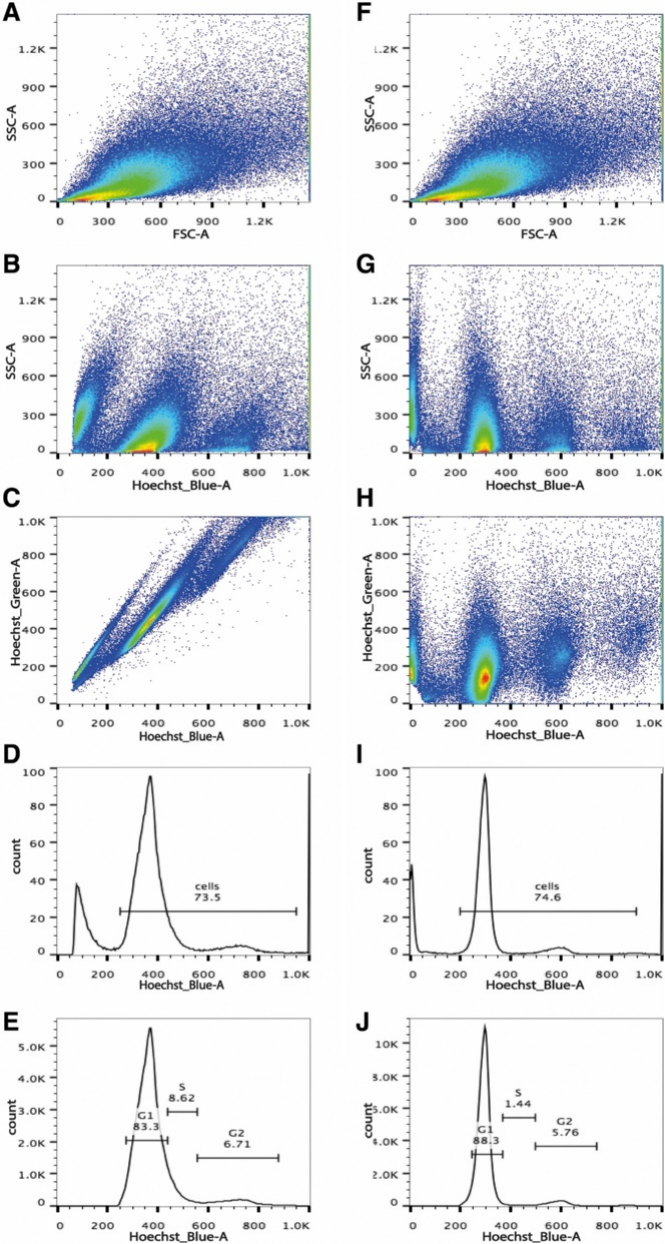
**Gating strategy for flow cytometry analysis of dissociated cells. A** & **F**. Density plot displaying Side scatter versus Forward scatter before **(A)** and after **(F)** compensation for autofluorescence. **B** & **G**. Representation of Hoechst staining in dot plots versus Side scatter before **(B)** or after **(G)** compensation for autofluorescence. **C** & **H** Representation of Hoechst staining in dot plots versus Hoechst Green signal before **(C)** or after **(H)** compensation for autofluorescence. The compensation breaks the correlation between SSC or Hoechst’s green signal with Hoechst’s blue signal. **D** & **I**. Representation of the cell cycle before **(D)** and after **(I)** compensating for autofluorescence. **E** & **J**. The first peak corresponds to G1 cycle cells, whereas the second peak to the G2 cycle cells, with cells in phase S in between. Compared to non- compensated sample **(E)**, compensation **(J)** enhances the precision of the cell cycle study.

The suspension contained cells of various sizes and structures, plus many cellular fragments and aggregates that would prevent proper gating using the classical FSC/SSC (size/structure) plot on a cytometer. It should be noted that debris resulting from mechanical dissociation of the kidney can lead to significant autofluorescence; this debris must be excluded in order to obtain a reliable and accurately quantitative signal. Because debris does not contain chromatin, Hoechst staining was used firstly to 1) detect DNA (real cells) and to exclude non-cellular debris, and 2) among intact cells, select singlets using a peak *versus* area plot (H/A, Additional file 1: Figure S1). Autofluorescence preponderantly emits in the green channels, which is why we used the V2 signal to compensate for the V1 signal, further excluding autofluorescent debris. This further improved the quantification of DNA cellular content, and made it possible to analyze the cell-cycle (Figure 2).

### Ex-vivo analysis of proximal tubular cells in the whole kidney suspension

Proximal tubular epithelial cells are highly differentiated renal cells. Prominin-1 is a surface glycoprotein expressed in the brush border [10-12]. Anti-prominin-1 antibodies are theoretically appropriate for use in that a) they are commercially available (anti-Prominin-1 rat monoclonal IgG1 antibody, clone MB9-3G8, Miltenyi Biotec, Bergisch Gladbach, Germany), b) they have been conjugated with a number of tags, and c) they target the extracellular region of prominin-1, which makes it possible to work with live, unpermeabilized cells.

**Figure 3:**
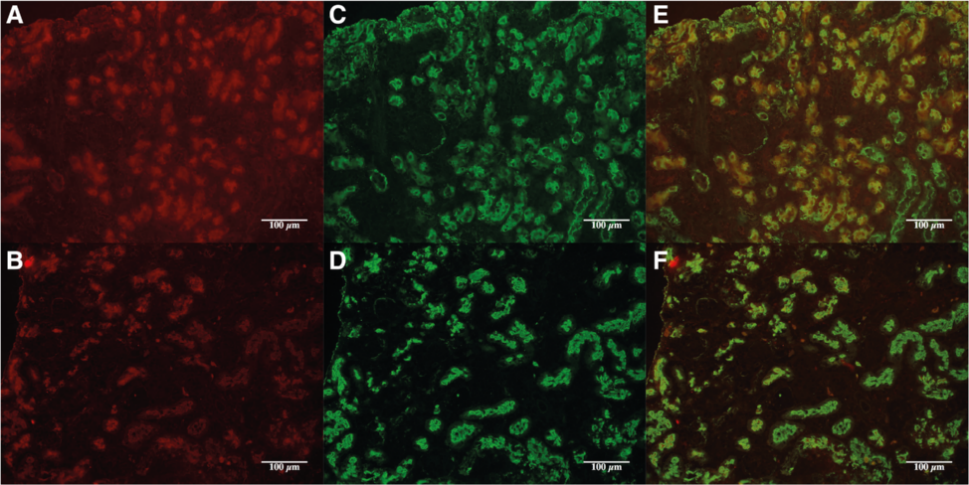
**Immunofluorescence analysis of control (A, C, E) and fibrotic kidneys (B, D, F) at magnification x20.** Prominin-1 (red) staining is shown in **A** and **B**, Megalin (green) staining is shown in **C** and **D**, and double staining is shown in **E** and **F**. Expression of megalin and prominin1 colocalize on the brush border of renal proximal tubular epithelium in mice. In fibrotic kidneys, tubules are interspaced with fibrosis but still express Prominin-1 and Megalin.

There are several isoforms of Prominin-1, and some anti-Prominin-1 antibodies show clone-dependent specificities for renal epithelial cellular subtypes [13]. To confirm that Prominin-1^+^ cells detected with clone MB9-3G8 were indeed proximal epithelial tubular cells, we measured its coexpression with megalin, a reliable marker of proximal tubules, by means of two different techniques. Using immunofluorescence and flow cytometry, we showed that Prominin-1 and Megalin colocalize (Figure 3 and Additional file 2: Figure S2 and Additional file 3: Figure S3). Megalin and Prominin-1 also colocalized after ischemia reperfusion injury (Figure 3 and Additional file 4: Figure S4).

**Figure 4:**
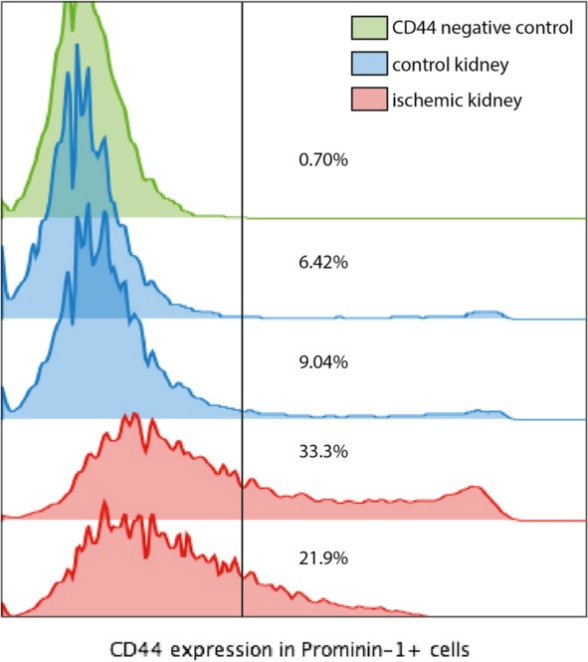
**Expression of CD44 by prominin-1** ^**+**^ **cells in a suspension of dissociated cells from control and ischemia reperfusion injured kidneys.** In green, cells are stained for prominin-1 but not for CD44 (negative control, 0.7% false positivity for CD44). In Blue, cells from control kidneys stained for Prominin-1 and CD44 (rare CD44+ cells). In red, cells from ischemia-reperfusion injury kidneys stained for Prominin-1 and CD44 (increased number of CD44+ cells).

We studied the effect of ischemia reperfusion injury on the expression of prominin-1 and CD44 (a marker expressed by injured renal epithelial cells) in cells from dissociated kidneys. In control mice, Prominin-1 was positive in 34.2 and 31.1% of the cells, and CD44 was expressed respectively in 6.42% and 9.04% of these Prominin-1^+^ cells. At day 2 post ischemia reperfusion injury, Prominin-1 was positive in 51.7 and 48.9% of the cells, and CD44 was expressed respectively in 33.3% and 21.9% of these Prominin-1^+^ cells (Figure 4).

### Isolation of proximal tubular cells from a whole kidney cell suspension

We reasoned that we could capture proximal tubular cells by using magnetic microbeads conjugated with an anti-prominin-1 antibody that specifically targets the proximal tubules; thus, a strong magnetic field applied to the preparation would separate the proximal tubular cells from the others. We therefore incubated the kidney cell suspension obtained as described above with microbead-conjugated anti-prominin-1 antibodies. Allophycocyanin (APC)-conjugated anti-prominin-1 antibodies were also added to label Prominin-1^+^ cells in order to check the quality of the magnetic separation by flow cytometry. We chose APC since its emission wavelenghth is barely affected by autofluorescence [14], and because it doesn’t overlap with Hoechst fluorescence spectrum.

**Figure 5:**
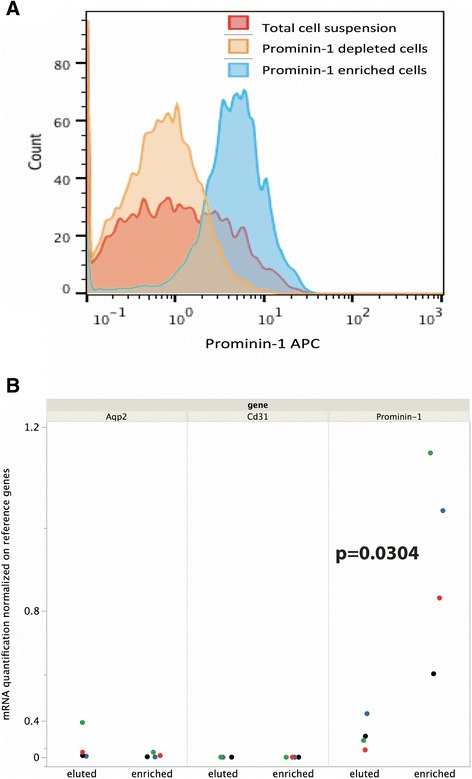
**Specificity of proximal tubular cells enrichment. A)** Density plot of prominin-1 expression showing an overlay of total kidney cell suspension in red (with a prominin-1 positive fraction estimated by flow cytometry at 37% of total cells in the suspension), prominin-1-depleted cells in orange (with a prominin-1 positive fraction estimated by flow cytometry at 15% of total cells in the suspension), and prominin-1 enriched cells in blue (with a prominin-1 positive fraction estimated by flow cytometry at 92% of total cells in the suspension). **B)** RT-PCR quantification of prominin-1, *Cd31* and *Aqp2* mRNAs in the eluted and the Prominin-1 enriched fractions. The results are shown as relative quantifications using the geometrical mean of gene *Gusb* and *Rpl32* as the reference.

The prominin-1 negative cells were eluted, and the Prominin-1^+^ cells were flushed at the end of the procedure after the magnetic field had been lifted. This isolation protocol is detailed in Table 2. The percentage of Prominin-1^+^ cells is assessed extemporaneously by flow cytometry (Figure 5A). Hundreds of thousand cells can be obtained per kidney. This yield may vary depending on experimental conditions (eg, 3.3 ± 0.5×10^5^, 2.0 ± 0.2 ×10^5^, and 4.2 ± 1.6×10^5^ respectively in control, day 2 and day 9 post ischemia reperfusion injury, Additional file 5: Figure S5). Assessed by flow cytometry, the purity of Prominin-1 cells in the positive fraction was 80.0 ± 11.1%. To further verify the specificity of the enrichment in proximal tubular cells, we used real-time PCR to compare the quantity of cell-type specific transcripts (using prominin-1 as a marker of proximal tubules, aquaporin 2 a marker of collecting ducts, and *Cd31* a marker of endothelial cells). We confirmed by RT-qPCR that the enriched fraction was indeed significantly enriched in Prominin-1 mRNA compared to the eluted fraction (p = 0.0304). *Cd31* and *Aqp2* mRNAs expression was very low in the enriched and the eluted fraction (Figure 5B).

**Table 2:** **Protocol for isolation of proximal tubular cells**

1.	Use the cell suspension obtained in Table 1.
2.	Add 20 μL of FcR blocking reagent.
3.	Mix well and incubate at 4°C for 10 minutes.
4.	Add 30 μL of anti-prominin-1 microbeads antibodies.
5.	Mix well and incubate at 4°C for 10 minutes.
6.	Add 10 μL of anti-prominin-1 APC antibodies.
7.	Mix well and incubate at 4°C for 5 minutes.
8.	Wash cells by adding 10 mL of dissociating buffer.
9.	Centrifuge at 500 g for 10 minutes.
10.	Aspirate the supernatant completely.
11.	Expand the cell pellet in 500 μL of dissociation buffer.
12.	Apply the solution to an LS column primed with 3 mL of buffer.
13.	Rinse the column with 3×3 mL of buffer, keeping the LS column away from the magnet.
14.	Collect the flow through solution and centrifuge at 500 g for 10 minutes.
15	Discard the supernatant and add 500 μL of buffer.
16.	Insert anew LS columns into an MACS separator magnet and prime it with3 mL of buffer.
17.	Apply the cell suspension obtained in 15.
18.	Wash the column with 3×3 mL of buffer.
19.	Remove the column from the separator and place it on a suitable collection tube.
20.	Flush the magnetically labeled cells with 5 mL of dissociation buffer.

### Cellular culture of a specific cell type isolated from a whole kidney cell suspension

**Figure 6:**
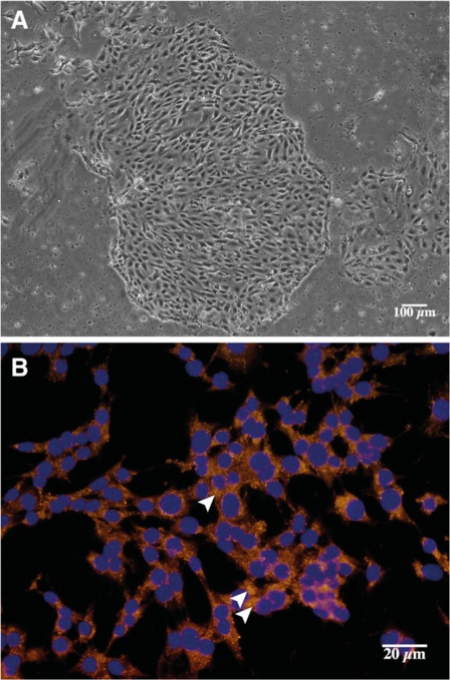
**Primary cultures of Prominin-1+ cells. A)** Contrast phase microscopy of Prominin-1+ cultured cells after 7 days. **B)** Immunofluorescence for ZO-1 in Prominin-1+ cultured cells. ZO-1 is expressed in the Prominin-1+ cultured cells, with a cytoplasmic pattern, enhanced at intercellular junctions (arrowheads).

**Figure 7:**
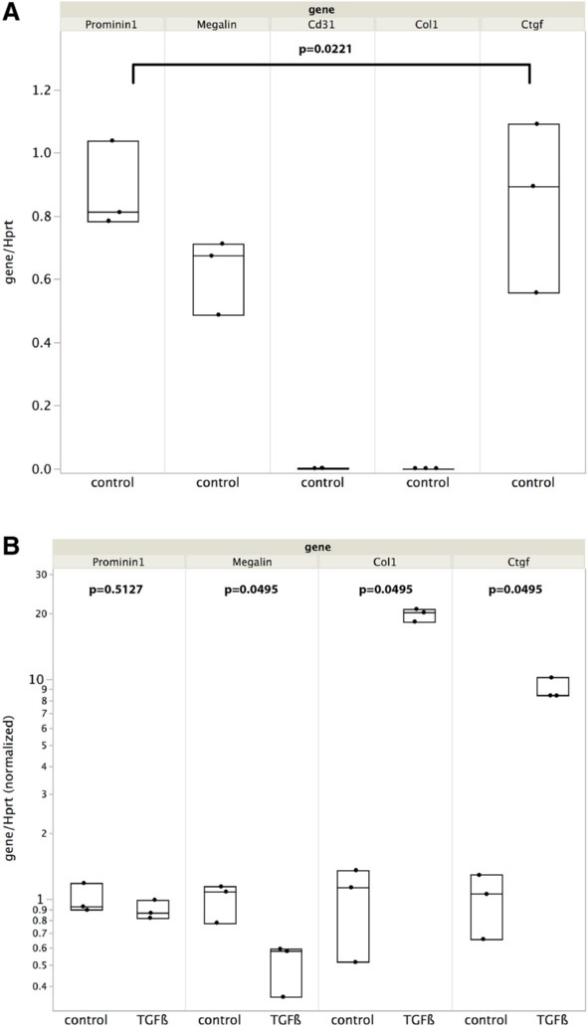
**RT-PCR analysis of Prominin-1** ^**+**^ **cultured cells. A)** Expression of *Collagen-1*, *Ctgf*, *Megalin*, *Cd31* and *Prominin-1* mRNAs in a primary culture of Prominin-1^+^ cells. *Prominin-1*, *Megalin* and *Ctgf* are strongly expressed as expected in proximal tubular cells, whereas *Collagen 1* and *Cd31* are virtually absent. **B)** Effect of TGFß on *Prominin-1*, *Megalin*, *Collagen-1* and *Ctgf* mRNAs expression. TGFß upregulated *Ctgf* and *Collagen-1* mRNAs, downregulated *Megalin* mRNA, and *Prominin-1* mRNA remained stable.

Prominin-1^+^ cells proved suitable for primary culture. After a period of proliferation, cells became contiguous and re-acquired an epithelial phenotype. Using contrast-phase microscopy, we observed a monolayer of polygonal, contiguous cells (Figure 6A). A component of epithelial tight junctions, the transmembrane protein Zona occludens 1 (ZO-1) is a marker of differentiated epithelia. ZO-1 was found to be expressed in Prominin-1^+^ cells (Figure 6B). The cells expressed mRNAs of *Prominin-1* and *Megalin*, and *Ctgf*, but not of *Cd31* or *Collagen 1* (gene expression levels differences among all group assessed by Wilcoxon test, p = 0.0221, Figure 7A). When exposed to TGF-ß, *Collagen 1* and *Ctgf* mRNAs were induced and *Megalin* mRNA was down-regulated (p = 0.0495 for each gene), whereas *Prominin-1* mRNA remained stable. These phenotypic changes are typical of the effect of TGF-ß on epithelial cells, and these data suggest that Prominin-1 expression remains stable at least in the first steps of epithelial injury (Figure 7B).

## Discussion

Our method can be used to analyze and separate distinct cell types from an organ (here, the kidney) during *in vivo* experiments. It yields operator-independent, quantitative and multi-parameter data on specific cell types. This resolves the issues of the irrelevance of cell cultures to investigate organ pathology and the cell heterogeneity characteristic of *in vivo* studies using whole organ lysates, which makes them unsuitable for proteomics or molecular biology.

Other approaches had been used previously: LASER micro-dissection on frozen or fixed tissues [15]; primary culture obtained by enzymatic kidney dissociation followed by FACS [16]; and, more recently, Translating Ribosome Affinity Purification (TRAP) [17].

Although these techniques can be useful, they all have some major drawbacks: 1) LASER microdissection can only be applied to dead cells (frozen or fixed, thus precluding cell culture) and cannot finely differentiate between cellular subtypes (usually, agross toluidine blue staining is performed to identify histological structures); 2) enzymatic dissociation/FACS implies a long and aggressive step of dissociation at 37°C when massive changes can occur within the cells [6,18], biasing any subsequent analysis; in addition, cell sorters are onerous equipment which are not suitable for benchtop use; 3) TRAP is restricted to the study of translation within cells, and needs a genetically modified strain for each cell type of interest.

Our method may a have some drawbacks. First, cell dissociation does not allow to study the interactions between different kind of cells. Second, it is dependent on the quality of the antibody used to capture epithelial cells. Third, the yield may vary depending on experimental settings, but this should be considered as informative data rather than as a bias.

In addition to allowing flow cytometry analysis and cell-type specific isolation, our methods yields live cells that can be used for cell-specific primary cultures, which may be very useful for studying the phenotype of a specific cell-type in any animal strain (especially genetically modified organisms in which no cell lines are available).

## Conclusion

Overall, we believe that our method offers a robust and simple tool to isolate and further study a specific cell type from an organ downstream of *in vivo* experimental models. This method allows a better understanding of cell-type specific biological processes in a solid organ, and provides easy cell-specific primary cultures for any chosen animal strain.

## Methods

### Tissue dissociation

Immediately after being harvested from adult c57bl6/J mice, each kidney was immersed in 1 mL of the dissociating buffer at room temperature. The renal capsule was removed and the kidney grossly chopped using a surgical blade. A cell dissociator (GentleMACS Dissociator, Miltenyl Biotec, California, USA) and dedicated tubes that preserve cell integrity (GentleMACS C-tubes, Miltenyl Biotec, California, USA) were used to dissociate cells in two sequential programs: a gentle program, followed by a stronger one, taking about 2 minutes at 4°C, with no added enzymes. Finally the supernatant was filtered through a 30-μm sieve. The dissociation buffer is made with 1X Phosphate Buffered Serum (PBS), 0.5% bovine serum albumin, and 2 mM EDTA.

### Analysis of a specific cell-type by flow cytometry, isolation of prominin-1^+^ cells and primary cell culture

Flow cytometry experiments were performed using the MacsQuant 10 VBR device (Miltenyi Biotec, Bergisch Gladbach, Germany). For DNA detection, Hoechst 33342 was used (Sigma Aldrich, France). The GentleMACS dissociator, GentleMACS C-tubes, anti-prominin-1 antibodies, LS columns (columns loaded with magnetic beads, Miltenyi Biotec, Bergisch Gladbach, Germany are suitable for a positive selection of magnetically labeled cells and FcR block (a solution of Fc immunoglobulin fragments designed to block non specific antibody binding by its Fc fragment to the Fc receptor expressed by some cells) were purchased from Miltenyi Biotec, Bergisch Gladbach, Germany. Full detail of the protocol is provided in Tables 1 and 2. Briefly, the cells obtained by mechanical dissociation were incubated with the proximal tubule specific anti prominin-1 antibody conjugated with magnetic microbeads. An anti APC –conjugated anti Prominin-1 antibody was then added to allow a fluorescence-based quality control of the isolation. The cells were then injected in the magnetic LS column, and rinsed in order to discard Prominin-1 negative cells in the flow-through, and to retain only Prominin-1^+^ cells. The LS column was then demagnetized by removing the magnet from the column, and the trapped Prominin-1^+^ cells were flushed in a collection tube. Cells were grown on 25 cm^2^ plastic flasks. The medium used for primary cell culture has been published elsewhere [19] and is detailed in the Additional file 6. For *in vitro* experiments, Prominin-1^+^ cells were stimulated with 10 ng/mL TGF-ß for 24 hours.

#### RNA extraction and RT-PCR analysis

RNA extraction was performed with RNA isolation COLUMNS (RNeasy micro, Qiagen). RT-PCR analysis was performed on a LightCycler 480 device using the maxima first strand cDNA synthesis kit (Fermentas) for reverse transcription, SybRGreen (Fast Start DNA Master Sybr Green I; Roche Applied Science, Roche Diagnostic) and PCR primers designed with the Roche Universal Probe Library as follows: *Aquaporin-2* TAGCCCTGCTCTCTCCATTG/GAGCAGCCGGTGAAATAGAT, *Prominin-1* GCCCAAGCTGGAAGAATATG/CAGCAGAAAGCAGACAATCAA, *Cd31* CGGTGTTCAGCGAGATCC/CGACAGGATGGAAATCACAA, *gusb* CTCTGGTGGCCTTACCTGAT/CAGTTGTTGTCACCTTCACCTC, *Rpl32,* GCTGCCATCTGTTTTACGG*/*TGACTGGTGCCTGATGAACT*, Ctgf*, TGACCTGGAGGAAAACATTAAGA/AGCCCTGTATGTCTTCACACTG, *Collagen1* GCAGGTTCACCTACTCTGTCCT*/*CTTGCCCCATTCATTTGTCT*, Megalin* TGGAGGATGCAGCCATATCT*/*GTGTGGACACTGGCACTCAG. RT-PCR was performed as follows: 95°C for 5 minutes, 45 cycles including 95°C for 15 seconds, 60°C for 15 seconds, and 72°C for 15 seconds. The melting curve was used to check the specificity of the PCR amplification (Additional file 7: Figure S6). The gene expression results were obtained by dividing the quantities of RNA for each gene by the geometrical mean of the quantity of RNA for the housekeeping genes.

#### Immunofluorescence

Immunofluorescence was performed on 3 μm thick slides for snap frozen kidneys, and on glass slides after 15 minutes fixation at −20°C with methanol for cultured cells. The slides were immersed in 1× PBS, incubated for 5 minutes in 1× PBS with 2% bovine serum albumin for blocking of non specific signal, then incubated with the primary antibody overnight at 4°C, washed 6 times in 1× PBS, incubated for 5 minutes in 1× PBS with 2% bovine serum albumin for blocking of non specific signal, incubated with the secondary antibody for 30 minutes at room temperature in the dark and washed 6 times in 1× PBS. For negative controls, the same protocol was used without incubating with the primary antibody. Immunofluorescence study for the detection of megalin and prominin-1 was performed using a sheep anti-megalin primary antibody (1/5000) [20] with an AlexaFluor 488 anti-sheep secondary antibody (1/1000, A11055, Invitrogen, Cergy Pontoise France); and a rat anti-prominin-1 primary antibody (1/25, MB9­3G8 Miltenyi Biotec, Bergisch Gladbach, Germany) with an AlexaFluor 546 anti-rat secondary antibody (1/1000, A11081, Invitrogen, Cergy Pontoise France).

#### Kidney injury models

Mice were housed in standard conditions in the Specific Pathogen Free animal facility of INSERM, UMR-S1155: 1284 L ventilated boxes with a 12/12 photoperiod, at a 20-24°C temperature with a 55+/−10% humidity. Food and water were available *ad libitum* The kidneys were harvested from male c57bl6/J mice after or sham surgery after a renal ischemia performed by clamping of the renal artery during 30 minutes followed by reperfusion for 2, 7 or 44 days. The surgery and the sacrifice were performed after anesthesia by intraperitoneal injection of penthotal. Animal care and the experimental protocol complied with the national and international guidelines and were approved by our local independent animal’s ethics institution (*Comité National de Réflexion Ethique sur l’Experimentation animal, numéro 5)* and the French Research Ministry (authorization number 00947.02). PG has an authorization for animal experimentations (N°A-75-1934).

#### Statistics

Comparisons between groups were performed using a Wilcoxon test. P-values ≤ 0.05 were considered as significant.

## Additional files

Additional file 1: Figure S1. A & B. Gating on nucleated cells after staining with Hoechst’s dye (A) and the resulting study population on a Forward scatter versus Side scatter dot plot (B). C & D. Gating on singlet cells by plotting the area under the curve by the peak of the Hoechst’s signal (C) and the resulting study population on a Forward scatter versus Side scatter dot plot (D). Additional file 2: Figure S2. A & B: Dot plot representing Side scatter versus Megalin – FITC. Cells positive for Prominin-1 are shown in red (A: negative control, B double stained sample). C & D: Dot plot of Side scatter versus Prominin1 – APC. Cells positive for Megalin are shown in green (C: negative control, D double stained sample). Prominin-1 and Megalin are expressed by the same cells (proximal tubular cells). Additional file 3: Figure S3. Controls for Prominin-1 (AlexaFluor 546, red) and Megalin (AlexaFluor 488, green) immunofluorescence analysis. A: double negative control acquired through the green filter (x20). B: double negative control acquired through the red filter (x20). C: Prominin-1 single-stained control acquired through the green filter to check the absence of emission of AlexaFluor 546 through the green filter (x20). D: Megalin single stained control acquired through the red filter to check the absence of emission of AlexaFluor 488 through the red filter (x20). E & G: Prominin-1 single-stained control (positive control) acquired through the red filter (E: x20, G: x60). F & H: Megalin single stained control (positive control) acquired through the green filter (F: x20, H: x60). Additional file 4: Figure S4. Masson’s Trichrome of the control (A, B) and fibrotic kidney (C, D) used for immunofluorescence in Figure 3, at magnification x20 (A, C) and x60 (B,D). Additional file 5: Figure S5. Number of cells obtained after isolation of Prominin-1^+^ cells in various experimental conditions. IRI: ischemia reperfusion. Additional file 6:
**Culture medium for primary culture of proximal tubular cells.**
Additional file 7: Figure S6. Melting peak curves *Gusb*, *Rpl32*, *Prominin-1*, *Megalin*, *Cd31*, *Aqp2*, *Collagen 1*, *Ctgf*. No unspecific amplification was detected.

## Abbreviations

RT-PCR: Reverse transcription polymerase chain reaction
DNA: Deoxyribonucleic acid
APC: Allophycocyanin
IgG: Immunoglobulin G
*Cd31*: *Cluster of differentiation 31*
*Aqp2*: *Aquaporin 2*
ZO-1: Zona occludens 1
LASER: Light amplification by stimulated emission of radiation
FACS: Fluorescence activated cell sorting
TRAP: Translating ribosome affinity purification
PBS: Phosphate Buffered Serum
*Gusb*: *Beta-glucuronidase*
*Rpl32*: *60S ribosomal protein L32*
