# Supplementary figures and images for: Ex vivo analysis of renal proximal tubular cells
Source: BMC Cell Biol. 2015 Mar 25;16:12. doi: 10.1186/s12860-015-0058-4 (PMC4379601; doi:10.1186/s12860-015-0058-4)

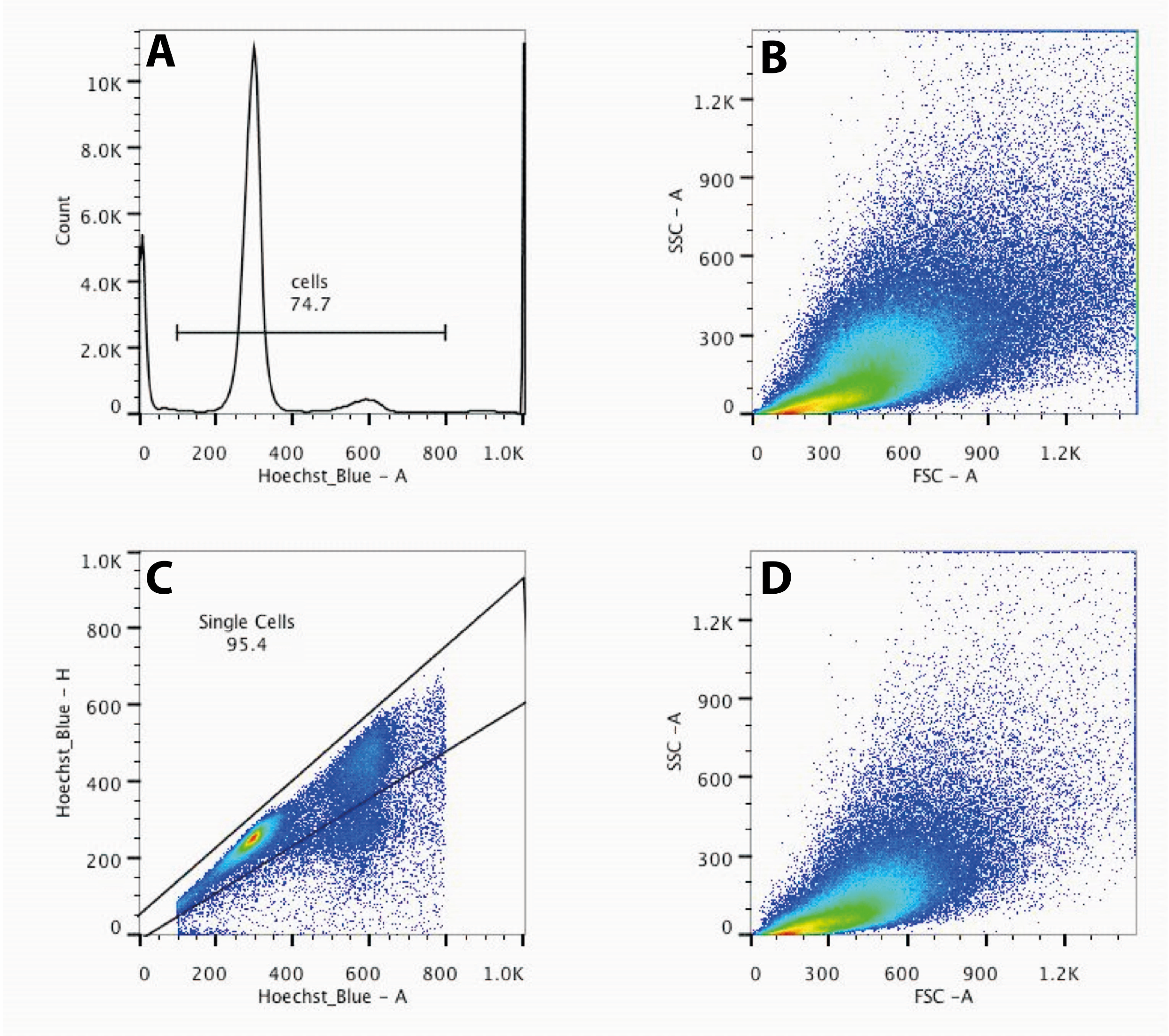

Supplement: Additional file 1: Figure S1. — A & B. Gating on nucleated cells after staining with Hoechst’s dye (A) and the resulting study population on a Forward scatter versus Side scatter dot plot (B). C & D. Gating on singlet cells by plotting the area under the curve by the peak of the Hoechst’s signal (C) and the resulting study population on a Forward scatter versus Side scatter dot plot (D). [file 12860_2015_58_MOESM1_ESM.png]

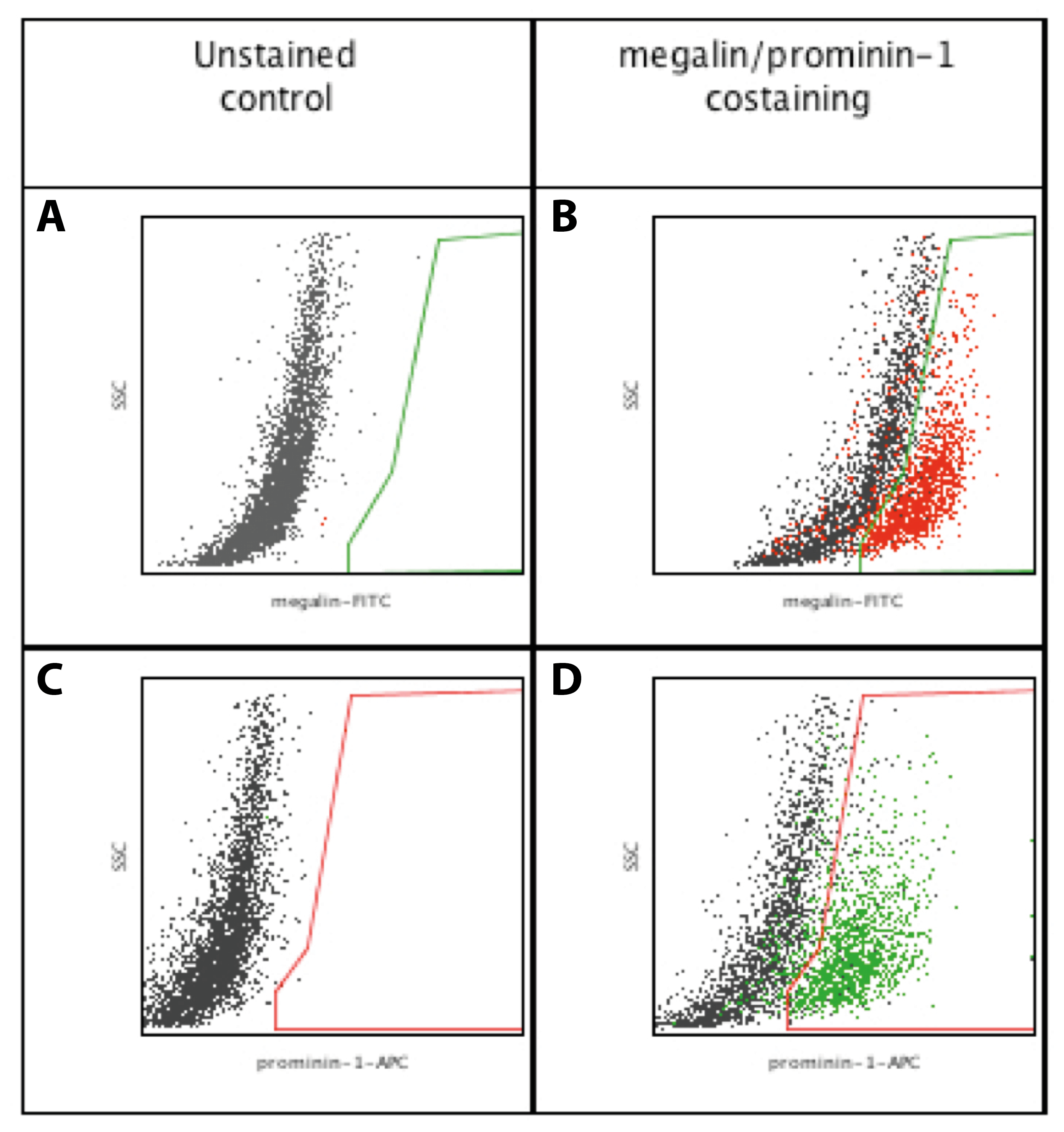

Supplement: Additional file 2: Figure S2. — A & B: Dot plot representing Side scatter versus Megalin – FITC. Cells positive for Prominin-1 are shown in red (A: negative control, B double stained sample). C & D: Dot plot of Side scatter versus Prominin1 – APC. Cells positive for Megalin are shown in green (C: negative control, D double stained sample). Prominin-1 and Megalin are expressed by the same cells (proximal tubular cells). [file 12860_2015_58_MOESM2_ESM.png]

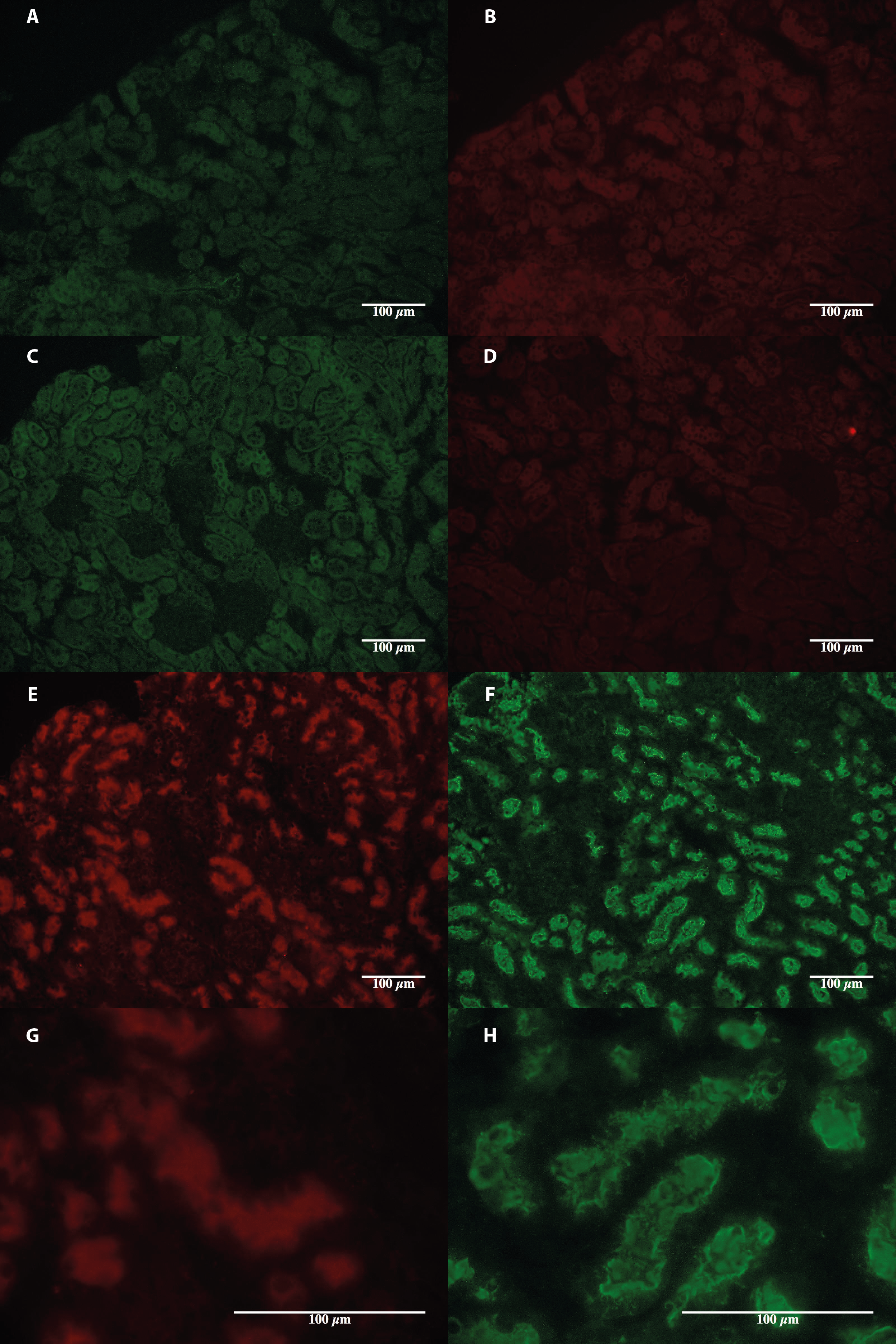

Supplement: Additional file 3: Figure S3. — Controls for Prominin-1 (AlexaFluor 546, red) and Megalin (AlexaFluor 488, green) immunofluorescence analysis. A: double negative control acquired through the green filter (x20). B: double negative control acquired through the red filter (x20). C: Prominin-1 single-stained control acquired through the green filter to check the absence of emission of AlexaFluor 546 through the green filter (x20). D: Megalin single stained control acquired through the red filter to check the absence of emission of AlexaFluor 488 through the red filter (x20). E & G: Prominin-1 single-stained control (positive control) acquired through the red filter (E: x20, G: x60). F & H: Megalin single stained control (positive control) acquired through the green filter (F: x20, H: x60). [file 12860_2015_58_MOESM3_ESM.png]

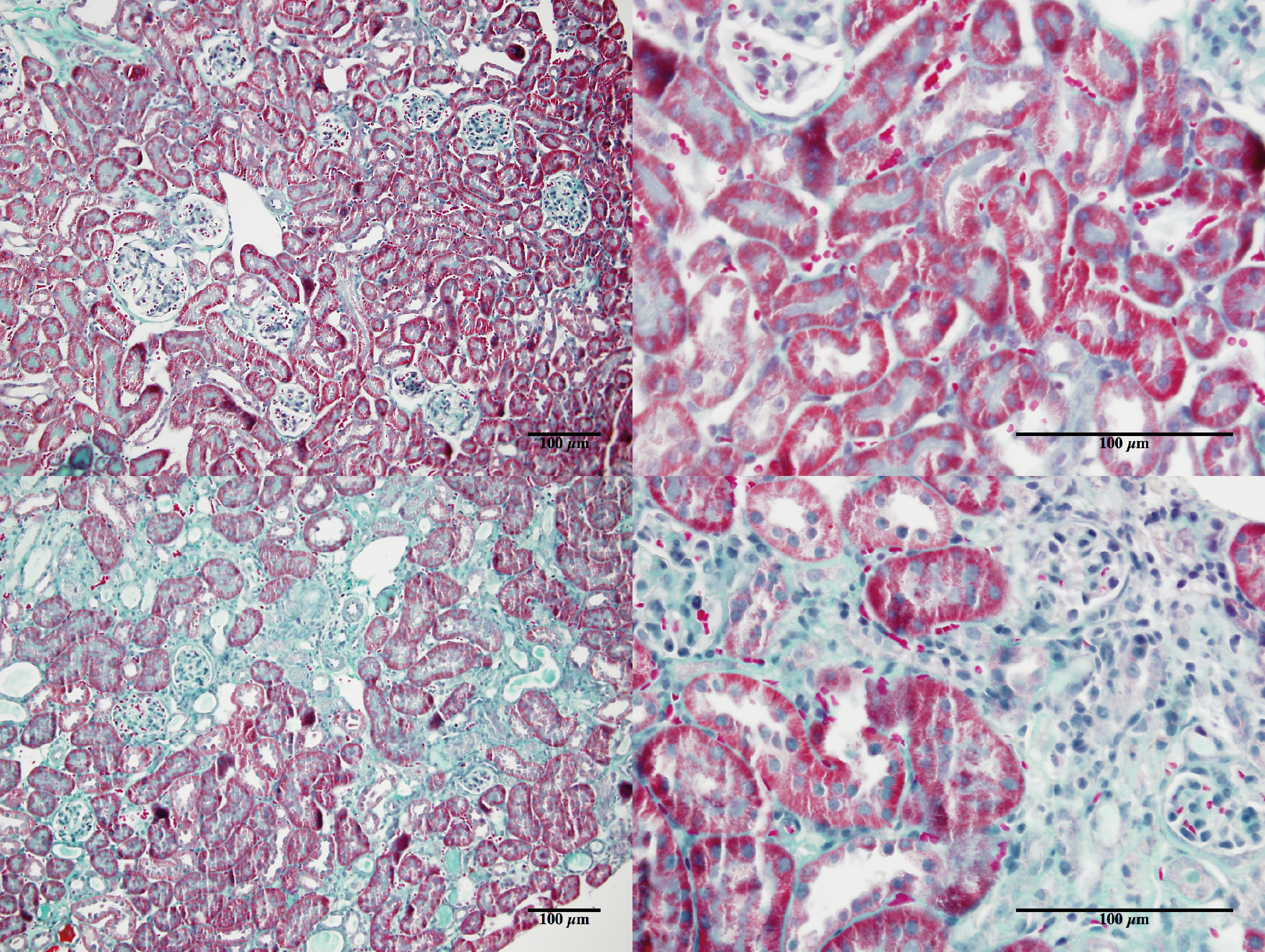

Supplement: Additional file 4: Figure S4. — Masson’s Trichrome of the control (A, B) and fibrotic kidney (C, D) used for immunofluorescence in Figure 3, at magnification x20 (A, C) and x60 (B,D). [file 12860_2015_58_MOESM4_ESM.png]

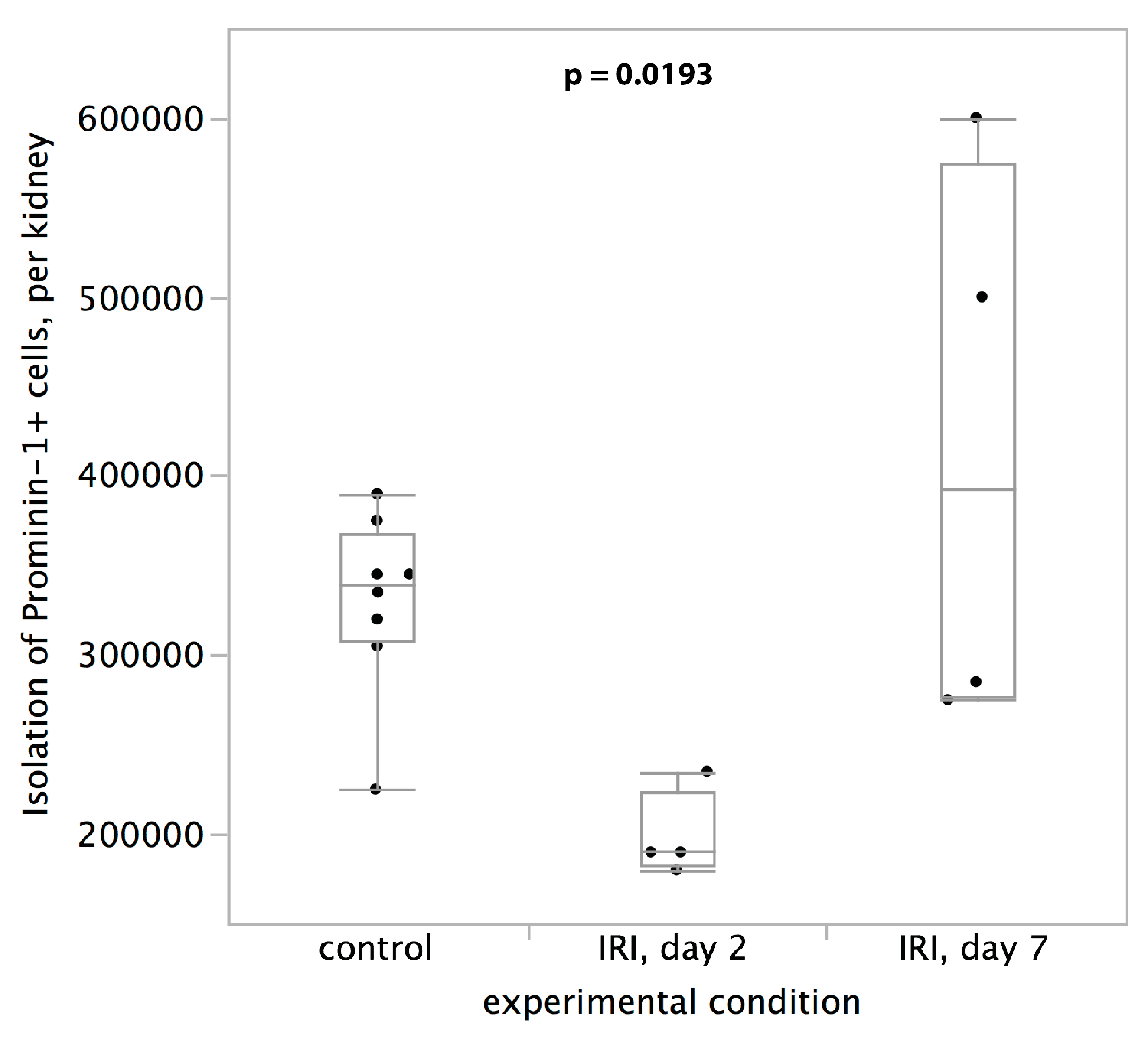

Supplement: Additional file 5: Figure S5. — Number of cells obtained after isolation of Prominin-1+ cells in various experimental conditions. IRI: ischemia reperfusion. [file 12860_2015_58_MOESM5_ESM.png]

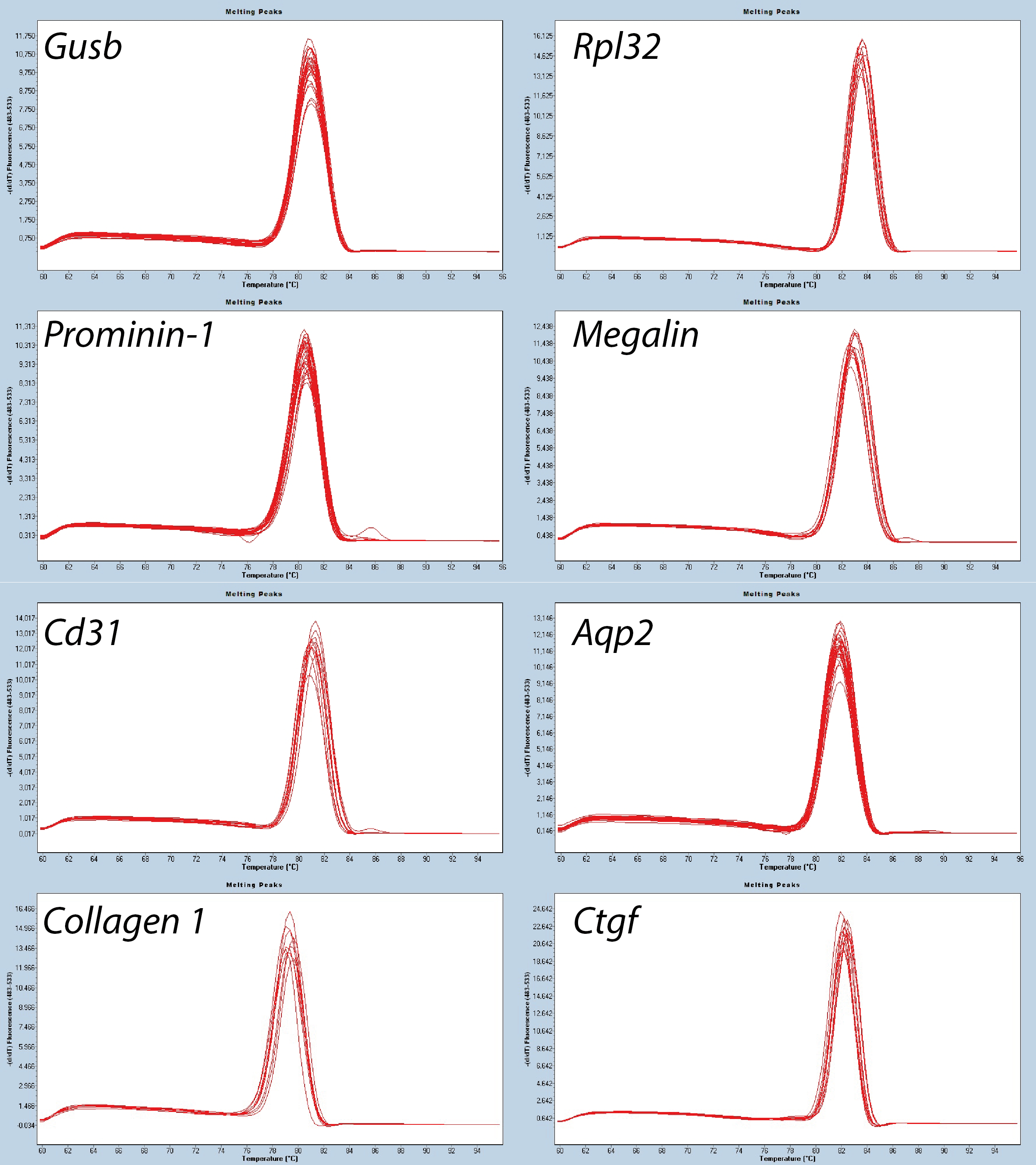

Supplement: Additional file 7: Figure S6. — Melting peak curves Gusb, Rpl32, Prominin-1, Megalin, Cd31, Aqp2, Collagen 1, Ctgf. No unspecific amplification was detected. [file 12860_2015_58_MOESM7_ESM.png]
