# Supplementary material for: Ex vivo analysis of renal proximal tubular cells
Source: BMC Cell Biol. 2015 Mar 25;16:12. doi: 10.1186/s12860-015-0058-4 (PMC4379601; doi:10.1186/s12860-015-0058-4)
Supplement: Additional file 6: — Culture medium for primary culture of proximal tubular cells. [file 12860_2015_58_MOESM6_ESM.docx]

**Additional file 6**

Culture medium for primary culture of proximal tubular cells.

Ham’s F12 nutrient mixture 50%, Dulbecco’s modified Eagle medium 50%

Insulin 5 µg/mL

Dexamethasone 5 x10^-8^M

Selenium 60 nM

Transferrin 5 µg/mL

Triiodothyronine 1 x10^-9^M

EGF 10 ng/mL

HEPES 20 mM

Glutamine 2mM

Decomplemented fetal calf serum 2%

D Glucose 0.2%
